# Evaluation of Effect of Oleuropein on Skin Wound Healing in Aged Male Balb/c Mice

**Published:** 2014-02-03

**Authors:** Fereshteh Mehraein, Maryam Sarbishegi, Anahita Aslani

**Affiliations:** 1 Minimally Invasive Surgery Research Center, Iran University of Medical Sciences, Tehran, Iran; 2 Department of Anatomy, Faculty of Medicine, Iran University of Medical Sciences, Tehran, Iran; 3 Department of Anatomy, Faculty of Medicine, Zahedan University of Medical Sciences, Zahedan, Iran

**Keywords:** Oleuropein, Skin, Wound, Aging

## Abstract

**Objective:**

Olive oil and olive leaf extract are used for treatment of skin diseases and wounds in Iran. The main component of olive leaf extract is Oleuropein. This research is focused on the effects of Oleuropein on skin wound healing in aged male Balb/c mice.

**Materials and Methods:**

In this experimental study, Oleuropein was provided by Razi Herbal Medicine Institute, Lorestan, Iran. Twenty four male Balb/c mice, 16 months of age, were divided equally into control and experimental groups. Under ether anesthesia, the hairs on the back of neck of all groups were shaved and a 1 cm long full-thickness incision was made. The incision was then left un-sutured. The experimental group received intradermal injections with a daily single dose of 50 mg/kg Oleuropein for a total period of 7 days. The control group received only distilled water. On days 3 and 7 after making the incision and injections, mice were sacrificed, and the skin around incision area was dissected and stained by hematoxylin and eosin (H&E) and Van Gieson’s methods for tissue analysis. In addition, western blot analysis was carried out to evaluate the level of vascular endothelial growth factor (VEGF) protein expression. The statistical analysis was performed using SPSS (SPSS Inc., Chicago, USA). The t test was applied to assess the significance of changes between control and experimental groups.

**Results:**

Oleuropein not only reduced cell infiltration in the wound site on days 3 and 7 post incision, but also a significant increase in collagen fiber deposition and more advanced re- epithelialization were observed (p<0.05) in the experimental group as compared to the control group. The difference of hair follicles was not significant between the two groups at the same period of time. Furthermore, western blot analysis showed an increased in VEGF protein level from samples collected on days 3 and 7 post-incision of experimental group as compared to the control group (p<0.05).

**Conclusion:**

These results suggest that Oleuropein accelerates skin wound healing in aged male Balb/c mice. These findings can be useful for clinical application of Oleuropein in expediting wound healing after surgery.

## Introduction

Natural remedies have been used for long time for prevention as well as treatment of minor diseases in Iran. Olive trees mostly are grown in the north of Iran, while Iranians use olive oil and olive leaf extract for treatment of skin diseases and wounds (1-3). The main component of olive leaf extract is Oleuropein which is rich in polyphenols, an antiinflammatory agent (4-6). As life expectancy is increasing and people live to their late 70 and 80 years. Prevention and treatment of the diseases in aged people may be more challenging as they are more vulnerable to diseases, and also they recover from them much slower than younger generations (7, 8). In aging process, cellular senescence, altered biosynthetic activity, as well as accumulation of oxygen species as a result of oxidative metabolism will increase in all organs of the body. All tissues in an aged body are more prone to adverse inflammatory reactions. Aging process in skin tissue involves changes in epidermis and dermis. The epidermis becomes thinner and atrophic. Also, the number of fibroblasts as well as their synthetic capacity will decrease significantly that indicates a reduction in matrix and collagen fibers of the dermis. These events ultimately lead to impaired wound healing process in aged skin (9-11). This research is focused on the effects of Oleuropein in expediting the wound healing process in aged skin.

## Materials and Methods

### Reagents

In this experimental study, Oleuropein was extracted from olive leaf in Razi Herbal Medicine Research Center (Lorestan, Iran). The air dried leaves powder was extracted with ethyl alcohol. The compounds were analyzed using High Performance Liquid Chromatography (HPLC) (12, 13). Primary antibody [anti vascular endothelial growth factor (VEGF)], alkaline phosphataseconjugated secondary antibody (goat polyclonal anti rabbit Ig G) and antiβ-actin mouse monoclonal antibody were supplied by Abcam, USA, while nitro-blue tetrazolium (NBT)/ 5-bromo-4-chloro- 3'-indolylphosphate p-toluidine (BCIP) tablets were purchased from Roche, Germany.

### Animals

Twenty four male Balb/c, 16 month old, with an average weight of 20-23 g were purchased from Iran Pasteur Institute, and housed in a temperature controlled room at 23 ± 2˚C. The animals were randomized equally into control and experimental groups. All animal works were approved by The Ethical Guidelines for the Care of Laboratory Animals of the Research Center of Iran University of Medical Sciences.

### Experimental design

Under ether anesthesia, the hairs on back of the neck of each mouse in both experimental and control groups were shaved and a 1 cm long fullthickness incision was made. The incision was left un-sutured. The experimental group received intradermal injections on both sides of the wound with a single daily dose of 50 mg/kg Oleuropein dissolved in distilled water for a total period of 7 days. The control groups received only distilled water. On days 3 and 7 after making the incision and injections, mice from each group were sacrificed, and the skin around the incision area was carefully dissected and divided into two sections. One section was fixed in 10% formalin in order to be stained by hematoxylin and eosin (H&E) and Van Gieson’s staining methods. The other section was lysed and used for western blot analysis. The fixed tissues were dehydrated in graded concentration of alcohol, cleared in xylene, infiltrated with paraffin, and finally, embedded in paraffin. Paraffin blocks of each animal were then cut into 5 μm thickness and stained with H&E to evaluate epithelialization which was scored between 0 and 3 for formation of new epithelial layer. The picture of each section was taken by a microscope (AX70, Olympus, Japan) equipped with a digital camera (Olympus, Japan) at ×400 magnification. The pictures were transferred to the computer using OLYSIA autobioreport software (Olympus optical co, Ltd, Japan); a grid was then superimposed on the picture, and percentages of cells with obvious nuclei in five separate microscopic fields were counted.

Van Gieson’s staining method was used for indication of type 1 collagen fibers by resulting in red color which was scored between 0 and 3 for type 1 collagen fiber deposition and epithelialization. Hair follicles in tissues of both groups stained by H&E method were counted randomly in five separate microscopic fields. The other section of skin tissues collected on days 3 and 7 post incision were homogenized in a lysis buffer and incubated on ice for 30 minutes. Lysates were collected after centrifugation (Eppendorf, Germeny) at 1300 rpm for 20 minutes at 4˚C. Protein concentration in the supernatant was determined using protein assay kit (Bio Rad, USA). The samples were separated by 12% sodium dodecyl sulfate polyacrylamide gel electrophoresis (SDS-PAGE) and transferred to nitrocellulose membrane (Millipore Corp. USA). The membrane was blocked for 1 hour in phosphate buffered saline (PBS) buffer containing 5% casein. The membrane was then incubated overnight with primary antibody for evaluation of vascular endothelial growth factor (VEGF, 1:400; Abcam, USA). The membrane was washed three times with tris buffer saline (TBS)-T, and incubated for 1 hour with secondary antibody (alkaline phosphatase-conjugated goat anti rabbit Ig G, 1:5000; Abcam, USA). Protein bands were detected using BCIP/NBT (Sigam, USA). The density of the bands was quantified using UVTec software (UVTec, UK).

The membrane was stripped and probed with β-actin (mouse monoclonal antibody; Abcam, USA). Statistical analysis was performed using Statistical Package for the Social Sciences (SPSS; SPSS Inc., Chicago, USA). The t test was applied to assess the significance of changes between control and experimental groups.

## Results

**Fig 1:**
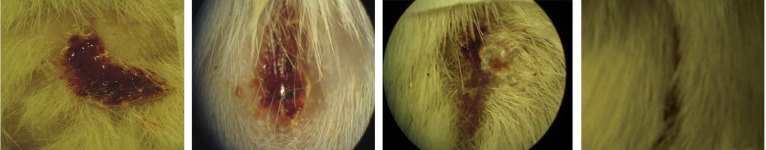
Effects of Oleuropein on wound healing on day 3 and 7 post incision compared to control groups. A, C; Control groups and B, D; Experimental groups.

**Fig 2:**
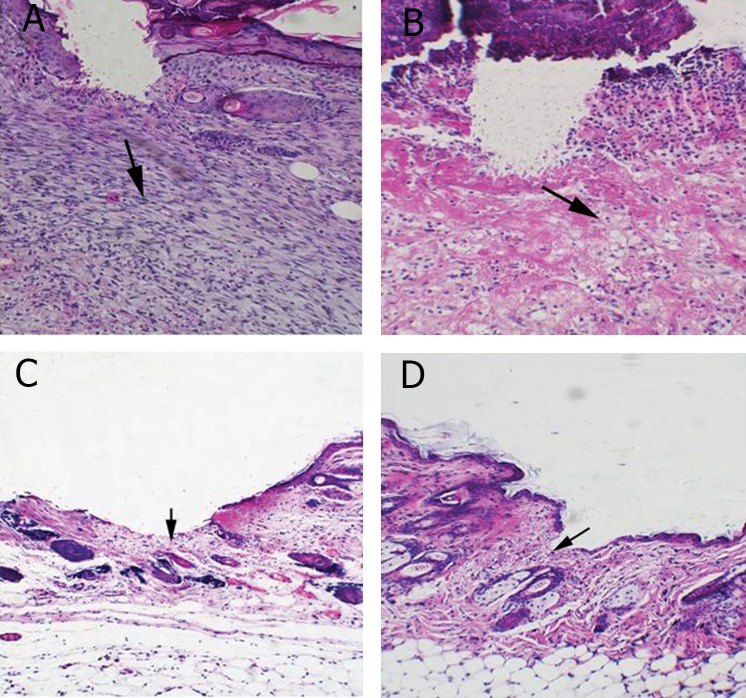
Histological analysis of H&E stained skin tissues from wounds on 3 and 7 days post incision. A. Cell infiltration increased in the wound site on day 3 post-incision in control group (arrow). B. In Oleuropein treated mice infiltrated cells were less than control group (arrow). C. Skin tissues from wound on day 7 post-incision in control group is shown, please note that epithelialization was not completed (arrow). D. Wound tissues from Oleuropein treated mice showed an advanced re-epithelialization (arrow) and dermal regeneration with significant decrease in cell infiltration compared to control group (Magnification ×200).

**Fig 3:**
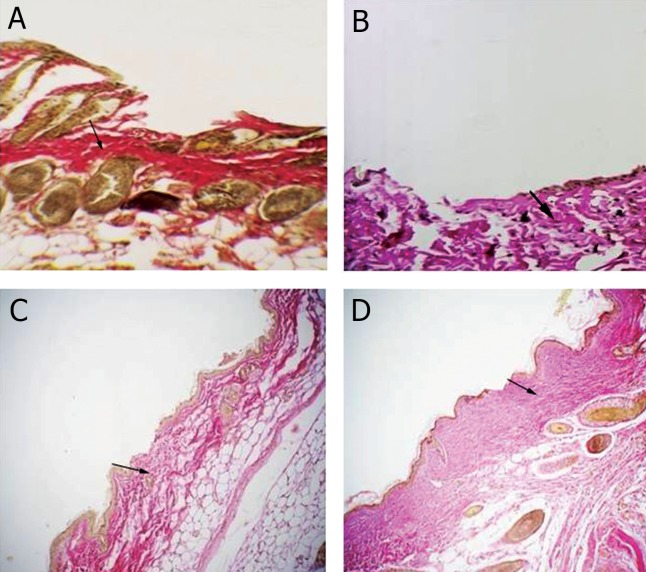
Van Geison’s staining for type 1 collagen fibers on 3 and 7 days post-incision in skin wound sections. A. Control group shows less and irregular arranged type1collagen fibers. B. Experimental group shows more collagen fibers. C. Skin wound section of control group on 7 day post-incision. D. Examination of incisional wound on day 7 in experimental group reveals dense and well aligned collagen deposition. The collagen fibers are shown by arrows (Magnification ×200).

**Fig 4:**
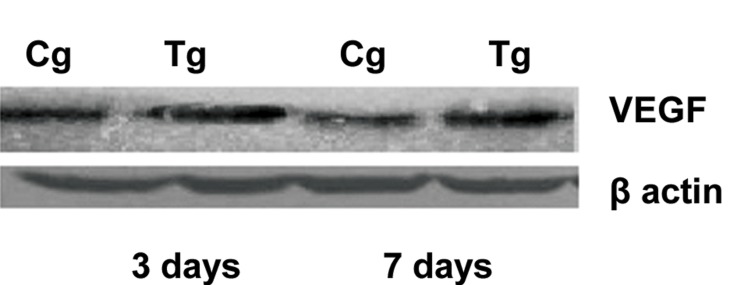
Western blot analysis of VEGF protein expression level from cell lysates of skin wound tissue from 3 and 7 days post incision. . Control group (Cg) and Oleuropein treated group (Tg). (Please see the text for details).

The mice in experimental group showed faster wound closure compared to control group on day 3 post incision (Table 1, Table Fig 1A, B). More advanced wound closure was observed in experimental group on day 7 post-incision, whereas in control group, the incision was not closed after the same period of time (p<0.05, Table 1,  Fig 1C, D). H&E staining of skin tissue from wounds showed an infiltration of cells in the control group on day 3 post incision (Fig 2A, Table 1); however, in the experimental group, the infiltrated cells were reduced significantly (p<0.05, Fig 2B, Table 1) and healing of the dermal damage was more noticeable. Re-epithelization was completed by day 7 post incision in experimental group as observed by improvement of wound healing compared to the control group (p<0.05, Fig 2C, D, Table 1). Van Gieson staining was used for demonstrating the type1 collagen fibers. Wound tissues in experimental group showed more collagen fibers than control group on day 3 (p<0.05, Fig 3A, B, Table 1). Morphometric analysis revealed that experimental group contained a higher content of type1 collagen fibers than control group on day 7 post incision, which are characterized by densely packed fibers (p<0.05, Fig 3C, D, Table 1). The differences of hair follicles were not significant between the two groups on days 3 and 7 post-incision (Table 1). Western blot analysis of different sections of wound tissues from experimental group revealed a higher level of VEGF protein on day 3 post-incision when compared to control group. The VEGF protein level in experimental group on day 7 post incision showed a significantly higher expression than in control group (p<0.05, Fig 4). The highest level of VEGF protein expression was observed on day 3 post incision in experimental group.

**Table 1:** Morphological parameters of the neck skin of control and experimental groups on days 3 and 7 post incision.


Analyzed parameters	Control group	Experimental group

**Epithelialization on day 3 (Score)**	0.01 ± 0.05	0.61 ± 0.1*
**Epithelialization on day 7(Score)**	0.4 ± 0.02	1.1 ± 0.05*
**Collagen fibers on day 3(Score)**	1.01 ± 0.02	1.6 ± 0.01*
**Collagen fibers on day 7(Score)**	1.02 ± 0.01	1.8 ± 0.03*
**Cell counting on day 3 (%)**	45 ± 0.1	30 ± 1.03*
**Cell counting on day 7 (%)**	26 ± 0.02	18 ± 0.01*
**Wound contraction on day 3 (%)**	19.2 ± 0.21	29.01 ± 1.03*
**Wound contraction on day 7(%)**	45.25 ± 0.32	55.30 ± 1.34*
**Number of hair follicle on day 3**	5.34 ± 0.22	5.5 ± 0.18
**Number of hair follicle on day 7**	6.1 ± 0.04	5.95 ± 0.3


* Significant p<0.05 All values are presented as mean ± SD

## Discussion

Altered inflammatory response, decreased collagen synthesis, delayed angiogenesis and slower re-epithelialization are observed in wound healing process in aged skin (4, 5). The effects of Oleuropein, the main constituent of olive tree leaf extract, on fibroblast culture showed a delay in senescence (13-16). Oleuropein, known as an antioxidant, reduces cellular damage to a minimum allowing improvement of wound healing. Another role of Oleuropein is the anti-inflammatory effect (17-20).

According to the present results, cell infiltration was reduced in Oleuropein treated wound tissues when compared to control group on days 3 and 7 post incision. The repair of wound tissue was more advanced in the experimental group than the control group. Fibroblasts are the main cells that are responsible for collagen synthesis. Type1 collagen fibers are the predominant feature of the skin that determines its tensile strength. Many studies have shown the role of increased collagen production in wound healing (18). In this study, Van Gieson’s staining of tissue sections from wound after incision showed that Oleuropein exerted positive effect on type1 collagen fiber synthesis. Overall, histological examination revealed that epithelialization and type1 collagen fiber content in experimental group is more significant when compared to the control group. The contraction of wound area showed a more rapid repair of the wound in Oleuropein treated group than the control group. An increase in tensile strength in experimental group may be due to the increase of type1 collagen fibers which confer strength to tissue and increase the rate of epithelialization (19). In this study the number of hair follicles was not significantly different between the two groups.

It seems that in short period of time Oleuropein failed to affect the hair follicles. Many reports showed the correlation of wound healing with stimulation of angiogenesis (21). In this study, we observed that intra-dermal injection of Oleuropein increases the VEGF level in experimental group on days 3 and 7 post-incision. VEGF is a pre-angiogenesis factor which promotes angiogenesis and wound healing. The effect of aging on angiogenesis in wound healing shows that angiogenesis is a major factor in wound healing in aged tissues, while the decrease of VEGF is responsible for impaired wound healing (21). The properties of Oleuropein will be studied more in our future projects, specially the molecular mechanisms of wound angiogenesis regulation and the interaction between extra cellular matrix and angiogenesis process contributing to wound healing.

## Conclusion

These results suggest that Oleuropein accelerates skin wound healing in aged male Balb/c mice. Based on these findings, Oleuropein may have clinical application in expediting wound healing after surgery.
